# Molecular and Physiological Variability in Bread Wheat and Its Wild Relative (*Aegilops tauschii* Coss.) Species under Water-Deficit Stress Conditions

**DOI:** 10.3390/biotech12010003

**Published:** 2022-12-28

**Authors:** Zahra Khodadadi, Mansoor Omidi, Alireza Etminan, Asa Ebrahimi, Alireza Pour-Aboughadareh

**Affiliations:** 1 Department of Biotechnology and Plant Breeding, Science and Research Branch, Islamic Azad University, Tehran P.O. Box 14515-775, Iran; 2 Department of Agronomy and Plant Breeding, Agricultural College, University of Tehran, Karaj P.O. Box 31587-77871, Iran; 3 Department of Genetic and Plant Breeding, Kermanshah Brunch, Islamic Azad University, Kermanshah P.O. Box 67146, Iran; 4 Seed and Plant Improvement Institute, Agricultural Research, Education, and Extension (AREEO), Karaj P.O. Box 31587-77871, Iran

**Keywords:** association analysis, genetic diversity, SSR markers, photosynthetic traits, wheat germplasm

## Abstract

*Aegilops* and *Triticum* spp. are two ideal gene pools for the breeding purposes of wheat. In this study, a set of Iranian accessions of *Aegilops tauschii* Coss. and *Triticum aestivum* L. species were evaluated in terms of some physiological and biochemical features under control and water-deficit stress conditions. Moreover, several simple sequence repeat (SSR) markers were employed to identify marker loci associated with the measured traits. The results indicated that water-deficit stress significantly affected all measured traits and the highest reductions due to water-deficit were recorded for shoot fresh and dry biomasses (SFB and SDB), stomatal conductance (Gs), leaf relative water content (RWC), and chlorophyll b content (Chl b). In molecular analysis, 25 SSR markers generated 50 fragments, out of which 49 fragments (98%) were polymorphic. Furthermore, the genetic variation observed within species is more than between species. The results of cluster and Bayesian model analysis classified all evaluated accessions into three main clusters. Under control and water-deficit stress conditions, 28 and 27 significant marker-trait associations (MTAs) were identified, respectively. Furthermore, 10 MTAs showed sufficiently stable expression across both growth conditions. Of these, the markers *Xgwm-111*, *Xgwm-44*, *Xgwm-455*, *Xgwm-272*, and *Xgwm-292* were associated with multiple traits. Hence, these markers could serve as useful molecular tools for population characterization, gene tagging, and other molecular breeding studies.

## 1. Introduction

Bread wheat (*Triticum aestivum* L.) is one of the most important cereals is grown almost all over the world. Based on FAO’s report from the year 2020, the global production of this crop is near 763 million tons [1]. As it turns out, wheat has huge germplasm compared with other crop plants and among its wild relatives, *Aegilops* species are the potential gene pools that have key roles in the evolution of both durum and bread wheat. *Aegilops tauschii* is a diploid goatgrass (2n = 2x = 14) and has a crucial role in wheat domestication by donating the D genome to bread wheat [2]. There are many pieces of evidence that show *Ae. tauschii* can be used as one of the main wild relatives of wheat in breeding programs due to its potential relative to various environmental stresses (for instance see [3]). Hence, it seems that this species can increase the motivation of wheat breeders to start various complementary studies. Among abiotic stresses, water deficit or drought considerably affects plant growth and restricts plant productivity in many regions of the world. It has been demonstrated that drought can negatively affect various morpho-physiological and biochemical mechanisms, which occur in all plant tissues, and also decreases yield performance [4,5]. Classical crop breeding improves plant productivity through various cycles, from crossing programs to selection of suitable genotypes with better phenotypes for the best traits, especially yield performance. The improvement of biomass is a key target trait for plant breeders, because improving yield performance is associated with the total plant above-ground biomass. Thus, it has been illustrated that the photosynthetic efficiency is a main physio-chemical parameter for net carbon gain. Hence, high productivity can be achieved through investigating the activity of photosynthetic apparatus and its related components, such as photosynthetic pigments, stomatal behavior, and chlorophyll fluorescence [6].

Chlorophyll is a critical molecule that is linked with the photosynthesis process, having important roles in light transformation processes. Further explanation of the mechanisms of chlorophyll fluorescence (CF) will have potential importance for yield improvement [7]. CF parameters including the initial fluorescence (Fo), maximal fluorescence (Fm), and maximum/potential quantum efficiency of photosystem II (PSII) (Fv/Fm), have been commonly used to detect differences in the activity of the photosynthetic apparatus in various plants under diverse growth conditions [4,8,9]. For instance, Pour-Aboughadareh et al. [9] revealed a significant correlation between the shoot dry biomass and Fv/Fm parameter in bread wheat and some *Aegilops* species under severe water-deficit conditions. Hence, such studies can provide information regarding the efficiency of PSII and also the association among CF parameters with other physio-chemical characters [10].

The discovery of new molecular markers associated with photosynthetic characters in wild relatives and landraces germplasm has important implications for bread wheat breeding. Progress in genomic tools and approaches aids breeders in the identification and selection of genomic regions controlling various agronomic and physiological traits. Among genetic approaches, association analysis is often used to discover the relationship between genotypic and phenotypic data [11]. Several benefits, such as much finer mapping resolution, providing broader genomic region coverage, and minimum confidence intervals of the detected loci have led to this approach being used as an alternative approach to classical mapping (QTL) in many crops. On the other hand, marker–trait association (MTA) analysis has become an important statistical tool for detecting genomic regions responsible for genetic traits. Furthermore, it is known as the ideal model in functional plant genomics and high-resolution mapping of QTLs [12].

Among the wild relatives of wheat, *Ae. tauschii* has an important role in wheat evolution and due to its enriched allelic variation has presented many valuable agronomic and physiological features common to wheat. Hence, identifying the genomic regions associated with various plant characteristics in this species can further highlight its breeding potential. Although numerous studies are available on the population genetic structure and association analyses in cultivated wheat and some wild relative species, there are few reports on physiological and photosynthetic property marker associations in bread wheat. Hence, this study aimed to (i) dissect the genetic diversity using physiological parameters under two growth conditions, (ii) evaluate the molecular diversity using several SSR markers, and (iii) disclose associations between some physiological parameters with the SSR markers.

## 2. Materials and Methods

### 2.1. Plant Materials and Growth Conditions

A set of accessions from *Ae. tauschii* (48 samples) and *T. aestivum* (47 samples) species was evaluated in this study. Further information about GenBank codes of accession is shown in Supplementary Table S1. After seed germination and seedling establishment, plants were arranged in a factorial experiment based on a randomized complete block design (RCBD) with three replicates in a research glasshouse under optimal photoperiod and temperature conditions. Each experimental plot consisted of a plastic pot (20 cm diameter and 40 cm height) containing five seedlings from each accession. In the stress condition, water deficit or drought treatment was applied based on field capacity (FC) methodology [13]. In this way, before applying the water deficit treatment, firstly, the FC of each pot was determined. Then, in each period of irrigation the stressed plants received water as much as 30% of their pot capacity. At the three-leaf stage of seedling growth, the stress treatment (FC = 30%) was initiated for 20 days.

### 2.2. Phenotypic Assessment

At the end of stress treatment, several physiological and biochemical traits were recorded from seedling plants. CF parameters including initial fluorescence (Fo), maximal fluorescence (Fm), maximum primary yield of PSII photochemistry (Fv/Fo), and maximum quantum yield of PSII (Fv/Fm) were calculated using a portable Optic-Science OS-30p Fluorometer (Opti-Sciences, Inc., Hudson, NH, USA) according to manufacturer’s guidelines. Portable Leaf Prometer (SC-1; Decagon Devices, Inc., Pullman, WA, USA) and chlorophyll meter (SPAD-502; Konic Minolta Sensing, Inc., Osaka, Japan) devices were employed to measure the stomatal conductance and relative chlorophyll content (SPAD), respectively. The chlorophyll a (Chl a), chlorophyll b (Chl b), and carotenoid (CAR) contents were measured as the photosynthetic pigments according to a protocol described by Lichtenthaler and Wellburn [14]. The leaf relative water content (RWC) was estimated as proposed by Blum [15]. To measure shoot fresh and dry biomasses, each seedling plant was harvested and weighed as fresh biomass (SFB). Then, the harvested samples were dried using an experimental oven (at 70 °C for 72 h) and weighed to estimate the shoot dry biomass (SDB).

### 2.3. Genotypic Assessment

The total genomic DNA was isolated from fresh leaves of five plants of each accession according to the CTAB protocol [16]. The quality of isolated DNA was tested using 2.5% agarose gel electrophorese. For the genotypic assay, a set of 25 primers were selected based on a list of designed SSR from Roder et al. [17]. All polymerase chain reaction (PCR) reactions were performed in 20 μL including 10 μL master mix 2XPCR (Ampliqon, Odense, Denmark), 2 μL template DNA from each sample, 6 μL double-distilled water, and 1 μL of each forward and reverse SSR primer. Amplification reactions were run as follows: 95 °C for 5 min, 35 cycles of denaturation at 95 °C for 45 s, primer annealing temperature for 45 s, and primer elongation at 72 °C for 1 min, and final extension at 72 °C for 5 min. PCR products were loaded on a 2.5% agarose gel, stained with safe view II, and visualized under UV light.

### 2.4. Statistical Analysis

For phenotypic data, an analysis of variance (ANOVA) was calculated on the basis of a factorial experiment using SAS software ver. 9.1 (SAS Institute, Cary, NC, USA). To study the effect of water-deficit stress treatment on measured traits, the relative change in each trait due to stress was calculated as used by Pour-Aboughadareh et al. [18]. Inter-relationships among the measured traits were investigated through a PCA (principal component analysis)-based biplot using XLSTAT software ver.5.01 (XLSTAT, Addisonsoft, Paris, France).

In the genotyping assay, all PCR products were scored as absent (0) or present (1). Based on the obtained binary data, the resolving power (Rp), polymorphism information content (PIC), and marker index (MI) were estimated as the three important informativeness parameters. To explain the distribution of molecular variation within and between *Ae. tauschii* and *T. aestivum* species, analysis of molecular variance (AMOVA) was calculated using the GenAlEx package ver. 6.5 [19]. Some genetic variation parameters, such as the number of observed (Na) and effective (Ne) alleles, Shannon’s information index (I), Nei’s gene diversity (H), and percentage of polymorphic loci (PPL), were estimated using the GenAlEx package. To investigate the grouping pattern of samples, a fan dendrogram was rendered based on Jaccard’s genetic similarities matrix using MEGA ver. 5.1 software [20]. Structure population analysis was carried out using STRUCTURE software version 2.3.4 [21]. The structure analysis was computed using a set of subpopulations (K = 1–10) in seven independent runs with 100,000 in the initial burn-in period and Markov Chain Monte Carlo (MCMC) iterations in each run. The optimum number of actual subpopulations (∆K) followed by the kinship data (K) were obtained using the STRUCTURE HARVESTER program [22]. Finally, the marker–trait association analysis (MAT) was computed through a mixed linear model (MLM) by incorporating phenotypic and genotypic data, the Q-matrix (Q), and the kinship matrix (K) using TASSEL ver. 2.1 [23].

## 3. Results

### 3.1. Phenotypic Variation

According to the results obtained from ANOVA, there was a significant difference observed between water-deficit treatments and also among accessions in terms of all measurements except CAR content among accessions (Table 1). The two-way interaction effect between accessions and water-deficit treatment main effects was significant for the SPAD index, Fo, Fv/Fm, Gs, Chl a, CAR, SFB, and SDB. Except for the Fo parameter, the mean values of all traits declined due to water-deficit stress when compared with the control conditions (Table 1). The results showed that water-deficit stress decreased SDW, Chl b, Gs, RWC, SFB, Chl T, Fv/Fm, Chl a, Fv/Fo, SPAD, and CAR by 70.27, 44.56, 38.58, 37.94, 34.41, 29.98, 22.98, 21.02, 16.19, 12.56, and 8.33%, respectively. On other hand, water-deficit treatment significantly increased Fo by 15.07% compared with control conditions.

To dissect the interrelationships among measured traits and also identify the best accessions in terms of multi-traits, the principal component analysis (PCA) was calculated based on both control and water-deficit stressed data. The results of this analysis showed that the first four PCs with eigenvalues 3.67, 1.91, 1.64, and 1.39 accounted for 71.78% of the total variation of physio-chemical properties under the control conditions (Table 2). The first PC accounted for 30.61% of the total phenotypic variation and was positively correlated with SPAD, Fo, Fv/Fm, SFB, RWC, Chl a, Chl b, Chl T, and Gs. The second PC justified 15.89% of the total variation and was mainly affected by SPAD, Fv/Fm, Fv/Fo, SFB, RWC, CAR, and Gs. The SPAD, Fo, RWC, and CAR showed a significant association with the third PC and with other measured traits explained 13.69% of the total variation. The fourth PC accounted for 11.60% of the total variation and was mainly associated with SPAD, Fv/Fm, Fv/Fo, Chl a, Chl T, CAR, and Gs.

On other hand, the results from the PCA for the water-stressed data showed that the first five PCs accounted for 79.04% of the total variation. PC1 accounted for 25.68% of the total variation and was positively associated with SPAD, Fo, Chl a, Chl b, Chl TSFB, RWC, and CAR. PC2 was mainly associated with SPAD, Fo, SFB, and RWC traits and generally accounted for 19.78% of the total variation existence in stressed data. Moreover, PC3 accounted for 15.49% of the total variation and it was correlated with SPAD, Fv/Fm, Fv/Fo, SFB, RWC, Chl a, Chl b, Chl T, and Gs. PC4 accounted for 9.23% of the total physio-chemical properties and was mainly influenced by SPAD, Fo, Fv/Fm, SFB, RWC, Chl a, and CAR. Finally, PC5 justified 8.86% of the phenotypic variation and was significantly influenced by Fo, SFB, SDB, RWC, Chl a, Chl T, CAR, and Gs (Table 2).

**Figure 1:**
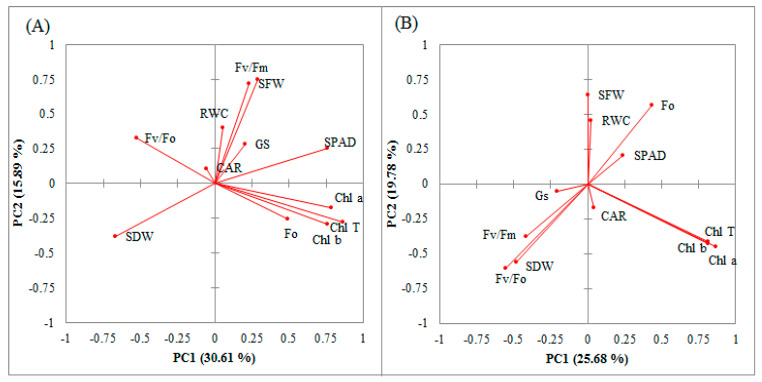
Biplots based on some photosynthetic-related parameters, physiological traits, and shoot biomass of the 95 investigated *Ae. tauschii* and *T. aestivum* species under control (**A**) and water-deficit stress (**B**) treatment. *SPAD* relative chlorophyll, *Gs* stomatal conductance, *Fv/Fm* maximum quantum yield of PSII, *Fv/Fo* maximum primary yield of PSII photochemistry, *Fo* initial fluorescence, *Chl a* chlorophyll a content, *Chl b* chlorophyll b content, *Chl T* total chlorophyll content, *RWC* leaf relative water content, *CAR* carotenoid content, *SFB* shoot fresh biomass, and *SDB* shoot dry biomass.

Because the first two PCs had the highest contribution in explaining the total phenotypic variation, the biplots were created based on PC1 and PC2. Under control conditions, there was a positive and significant correlation among Gs, CAR, RWC, SFB, Fv/Fm, Fv/Fo, and SPAD. Correlations among SPAD, Chl a, Chl b, Chl T, and Fo were also positive and significant. In this condition, SDB only positively correlated with Fv/Fo (Figure 1A). On the other hand, there was a different pattern of association among measured traits under water-deficit stress conditions. As shown in Figure 1B, there was a positive and significant correlation among Gs, CAR, Fv/Fm, SDB, and Fv/Fo traits. Correlations between SFB and RWC, Fo and SPAD, and SPAD and RWC and SFB were positive and significant. All photosynthetic pigments (Chl a, Chl b, Chl T, and CAR) indicated a significant and positive correlation with each other.

### 3.2. Genotypic Variation

In total, 50 fragments were amplified using 25 SSR primers, out of which 49 fragments (98%) were polymorphic (Table 3). The polymorphism information content (PIC) value varied between 0.14 (*Xgwm-232*) and 0.38 (*Xgwm-624*) with an average of 0.32. The marker index (MI) value ranged from 0.28 to 0.98 with a mean of 0.80, and the markers *Xgwm-232* and *Xgwm-121* showed the minimum and maximum values, respectively. The Rp value, with an average of 1.27, varied between 1.02 (*Xgwm-608*) and 1.88 (*Xgwm-232*). The gene diversity (H) value, with a mean of 0.41, ranged from 0.15 (*Xgwm-232*) to 0.50 (*Xgwm-469*, *Xgwm-583*, *Xgwm-608*, and *Xgwm-157*).

**Figure 2:**
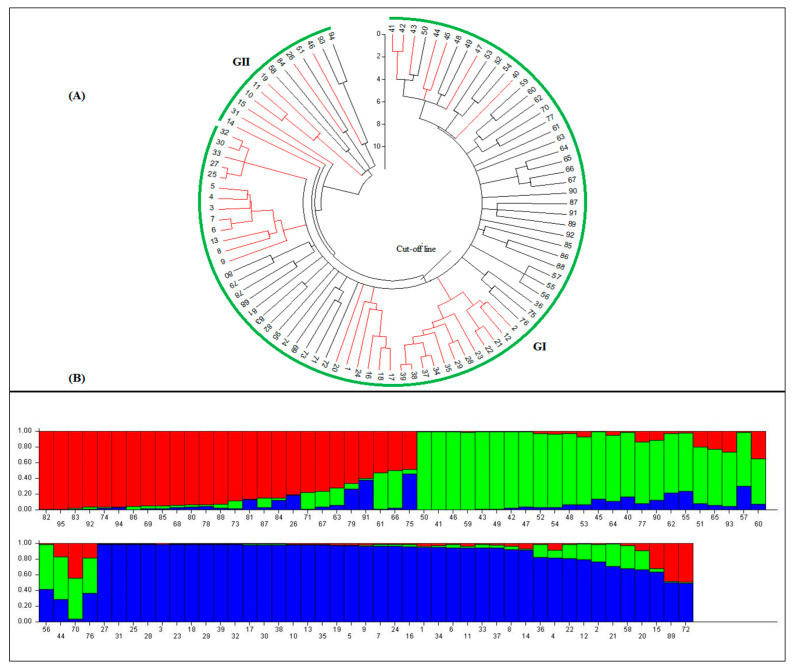
(**A**) The fan dendrogram generated using the neighbor-joining clustering method based on the 95 investigated samples. The red and black lines indicate *T. aestivum* and *Ae. tauschii* accessions, respectively. (**B**) Population structure of the 95 investigated individuals using 24 polymorphic SSR markers inferred by structure. The actual number of subpopulations (K = 3) was estimated according to Evanno’s test [24]. In both panels, numbers indicate the investigated accessions (Supplementary Table S1).

Based on the results of the analysis of molecular variance (AMOVA), the percentage of molecular variance was higher within species than among them (Table 4). Moreover, the genetic variation parameters revealed the highest values for the observed (Na) and effective (Ne) number of alleles, Nei’s gene diversity (He), Shannon’s information index (I), and percentage of polymorphic loci (PPL) were estimated for *Ae. tauschii* compared with *T. aestivum* species (Table 4). The cluster analysis was calculated using the neighbor-joining (NJ) algorithm and indicated an unclear grouping pattern among the 95 investigated accessions. The rendered fan dendrogram displayed that all accessions were clustered into two main groups so that each group embraced samples from each species (Figure 2A).

### 3.3. Population Structure and Marker-Trait Association

A genotypic data matrix that was generated using SSR markers was used for determining the population structure of the investigated accessions using the Bayesian clustering model. According to the results, the optimum values of ∆K was estimated as K = 3; therefore, all investigated accessions were placed into three distinguished sub-populations with 23, 26, and 39 members, respectively (Figure 2B). Each sub-population consisted of different accessions from both species. However, seven accessions showed a high range of admixes coefficient (>0.5) and were separated from other individuals. The marker–trait association analyses between SSR data and the physio-chemical characteristics were performed using an MLM method for both the control and water-deficit stress conditions, separately.

The MTA analysis identified that 28 markers were associated with measured traits under the control conditions. Of these, nine markers were associated with more than one trait. The coefficient of determination (*R*^2^) ranged from 5.27 to 16.91%. Based on the results, the *Xgwm-111* marker showed a simultaneously significant linkage with Chl a and Chl b contents. Furthermore, the association between *Xgwm-271* and CAR content and the Fo parameter was significant. The marker *Xgwm-272* showed a significant association with Chl T content, Fv/Fm, and Gs. The marker *Xgwm-292* showed a significant association with Fv/Fm and Fv/Fo parameters. Moreover, for Chl a and Chl b, a significant association was found with the *Xgwm-44* marker. The marker *Xgwm-455* showed simultaneous significant association with Chl a, Chl b, and Chl T contents, and SFB. Furthermore, *Xgwm-484* indicated a significant association with RWC, Chl b content, SFB, SDB, and FV/Fo. Two markers, *Xgwm-565* and *Xgwm-582,* were associated with SPAD and SDB, simultaneously. However, the *Xgwm-16*, *Xgwm-232*, and *Xgwm-325* markers showed a significant association with Gs, RWC, and Chl T content, respectively (Table 5).

Under water-deficit stress conditions, 27 significant MTAs were found. Of these, six markers were associated with more than one trait. The *R*^2^ index ranged from 5.24 to 15.55%. The marker *Xgwm-111* displayed a significant association with SPAD and Chl a, b, and T contents, simultaneously. Similarly, the marker *Xgwm-271* indicated a significant association with Fo and CAR. The traits Fv/Fo, Gs, and Fv/Fm were significantly associated with the *Xgwm-272* marker. The marker *Xgwm-292* exhibited a simultaneous significant association with the Fv/Fo, RWC, and Fv/Fm traits. Moreover, the association between *Xgwm-44* and chlorophyll components was significant. Likewise, the *Xgwm-455* marker had a significant association with chlorophyll components. In addition to these associations, CAR content showed a significant association with the *Xgwm-121* marker. Moreover, associations between RWC and two markers, *Xgwm-296* and *Xgwm-301,* were significant. The SFB showed a significant association with the *Xgwm-232* and *Xgwm-484* markers. Furthermore, the association between SDB and the *Xgwm-565* and *Xgwm-582* markers were significant.

## 4. Discussion

Among the environmental stresses, water deficit or drought is known as the most determinative factor for the growth and productivity of crop plants. Hence, the development of new varieties tolerant to drought stress is one of the most promising strategies for improving yield performance in wheat breeding programs. Screening of plant genetic materials for tolerance to water-deficit stress either should be fast, easy, non-destructive, and inexpensive. Moreover, the used tools for this task should allow researchers to take several measurements from a single plant. Hence, greenhouse screening methods, especially at early growth stages can be useful to accelerate identifying tolerant plant materials [25]. Bread wheat, as one of the most important cereal crops, has a key effect on food security in the world. The knowledge of genetic diversity in this crop opens new windows regarding the impact of physiology research in future breeding programs. The current study dissected the physio-chemical variation, genetic diversity, population structure, and association between several microsatellite markers with several physiological and photosynthetic properties in the diverse germplasm of bread wheat and its wild relative *Ae. tauschii* germplasm. *Ae. tauschii* is one of the most important wild relatives and as a D genome donor directly contributed to the genomic constitution of bread wheat. In addition, this species is known as a perfect gene pool for wheat breeding programs with the aim of improving the genetic background of bred varieties to withstand various environmental stresses [9].

According to our obtained results, the phenotypic responses of investigated accessions under control and water-deficit stress conditions revealed a considerable level of genetic variability (Table 1). Under stress conditions, all of the measured traits (except Fo) indicated a degree of reduction between 8.33 (in CAR content) and 70.27% (in SDB). Specifically, shoot fresh and dry biomasses, stomatal conductance, RWC, and Chl b content showed the largest reduction (Table 1). This result is in agreement with Pour-Aboughadareh et al. [9], who reported a high effect of water-deficit stress on shoot biomass and some physiological traits in a set of wheat genotypes and its wild relatives. Among the physiological and biochemical processes, photosynthesis and its related pigments are sensitive to drought conditions. In other words, the optimal concentrations of photosynthetic pigments and the health of the photosynthetic apparatus have critical roles in maintaining plant growth and development [17,26,27,28,29,30]. It has been reported that chlorophyll components are susceptible to water deficiency and can affect the yield performance and even the grain quality in wheat [26]. Therefore, the genotypes with a high concentration of photosynthetic pigments may show a relative tolerance to water-deficit stress. In the present study, our results showed that stress treatment declined total chlorophyll content and each of its components (Chl a and Chl b) by more than 20% compared with the control treatment (Table 1); this finding is in agreement with previous studies [18,26]. However, the reduction of CAR content is not significant (~8%). It is worth noting that CAR further plays a role as an antioxidant. Hence, it may help plants to tolerate drought stress by scavenging the reactive oxygen species (ROS) [31]. Moreover, our findings revealed that water-deficit stress negatively affected CF parameters and stomatal conductance. In addition to this result, we found that there was a considerable genetic variation among the investigated accessions for these traits.

In general, similar to other agronomy traits, physio-chemical properties and physiological traits commonly indicate a high level of phenotypic variability [32]. Multivariate methods provide efficient tools to capture phenotypic variation and dissect interrelationships among different traits [12]. In this study, PCA analysis indicated that the measured traits captured a large portion of the total phenotypic variation (71.78 and 79.04% in the control and water-deficit treatments, respectively), revealing that the measured traits were effective in evaluating the association among the measured traits (Table 2). Under both growth conditions, all photosynthetic pigments positively correlated with shoot fresh biomass. Additionally, the association among Fv/Fm, Fv/Fo, Gs, and CAR traits were positive and significant (Figure 1A). However, under water-deficit stress conditions, shoot dry biomass was positively correlated with Fv/Fo, Fv/Fm and Gs (Figure 1B). Because the Fv/Fo and Fv/Fm parameters showed positive correlations with shoot biomasses in both growth conditions, they could be used as screening tools to identify good accessions with high capability in terms of photosynthetic activity [10].

As another part of the results, the high percentage of polymorphism (98%) detected via SSR markers suggests that the used primers could be employed as powerful tools for discovering the molecular variability in the investigated accessions (Table 3). Most of the primers used showed the highest percentage of polymorphism, as well as high values for PIC, MI, and Rp indices. The results of AMOVA indicated that the rate of genetic variability within species is more than between them, suggesting that the gene flow among species is limited and each of them has a diverse genetic background (Table 4). As a part of the results, *Ae. tauschii* accessions showed higher genetic variation parameters relative to *T. aestivum* accessions (Table 3). Indeed, this result is in agreement with other studies, where Naghavi et al. [33,34] and Pour-Aboughadareh et al. [35] reported considerably higher levels of genetic variation among *Ae. tauschii* than *T. aestivum* accessions.

In the present research, the population structure was deciphered by a Bayesian clustering algorithm. The findings showed that all samples were separated into three actual sub-populations (Figure 2). Deciphering the association between phenotypic and genotypic data depends on the used statistical model [36,37]. In this regard, the mixed linear model (MLM) is widely used to detect marker–trait associations. The results of association analysis for photosynthetic pigments, CF parameters, and some physiological traits revealed that the MLM model was effective in detecting significant MTAs. This result was confirmed by the findings reported by Mehrabi et al. [12], who stated that the MLM model is more efficient in detecting significant MTAs in germplasm materials. In the present work, a total of 28 and 27 significant MTAs were detected in the control and water-deficit stress conditions, respectively (Table 5). Under both treatments, the explained *R*^2^ values for the identified MTAs were high (range 5.27–16.91% in the control and range 5.24–15.55% in the stress conditions), indicating that many genes contributed to the main part of the quantitative trait [12]. However, some MTAs indicated a lower variation (*R*^2^ ≤ 10%); hence, this may be explained by the fact that these markers reveal minor effects on the measured traits [38]. Under control conditions, *Xgwm-484*, *Xgwm-445*, and *Xgwm-272* were identified markers that were associated with the majority of measured traits such as SFB, SDB, Fv/Fo, Fv/Fm, and RWC. On other hand, under water-deficit stress conditions, six markers were associated with the various measured traits (Table 5). Our results revealed that some MTAs were similar under two water treatments, suggesting the environmental conditions are not effective in these associations. Indeed, these results indicated that different genes might contribute to the same traits in different conditions. In the present study, 10 markers indicated a stable association with different traits under both growing conditions, notably the markers *Xgwm-111*, *Xgwm-44*, and *Xgwm-455* with Chl a content; *Xgwm-111* and *Xgwm-44* with Chl b content; *Xgwm-121* and *Xgwm-271* with CAR content; *Xgwm-272* with Gs; *Xgwm-271* with Fo; *Xgwm-455* with Chl T; *Xgwm-272* and *Xgwm-292* with Fv/Fm; *Xgwm-292* with Fv/Fo; *Xgwm-484* with SFB; and *Xgwm-565* and *Xgwm-582* with SDB.

## 5. Conclusions

Screening plant genetic resources for environmental stresses has a critical role in identifying tolerant plant samples. In this regard, deciphering the association between genomic regions and phenotypic data can accelerate breeding programs. The present study revealed a high level of genetic diversity among evaluated wheat germplasm. Although water-deficit stress treatment decreased all measured traits, some biochemical traits, such as Fv/Fo and Fv/Fm parameters, showed significant associations with shoot biomass. Hence, these parameters can contribute to per-screening programs with goal of identification of drought-tolerant accessions at the early growth stage. Moreover, the used SSR markers showed a high degree of efficiency in detecting polymorphism and genetic diversity within species and were also a powerful tool for identifying significant MTAs. At the species level, the highest values of genetic variation parameters were estimated for *Ae. tauschii* species. Hence, this species can be considered as a source of variation for the discovery of new drought-tolerance genes. Taken together, our results can contribute to completing knowledge regarding the genetic basis of physiological- and photosynthetic-related parameters. Moreover, our findings will play a critical role in the conservation and management of Iranian wheat germplasm.

## Figures and Tables

**Table 1:** Analysis of variance (ANOVA) and mean values for measurements of physiological and biochemical traits across the 95 investigated *Ae. taushii* and *T. aestivum* accessions.

Traits	Replication (df = 2)	Stress (S; df = 1)	Accession (A; df = 94)	S×A (df = 94)	Error (df = 378)	MC ^†^	MS ^†^	RC ^†^
Relative chlorophyll	120.94	3169.03 ***	64.12 ***	34.32 ***	15.91	37.62	32.90	12.56
Initial fluorescence	0.001	0.019 ^ns^	0.001 ***	0.0003 ***	0.0002	0.08	0.09	−15.07
Maximum quantum yield of PSII	0.076	4.93 ***	0.005 ***	0.003 **	0.003	0.81	0.62	22.98
Maximum primary yield of PSII	62.72	57.54 ***	1.70 *	1.16 ^ns^	1.23	3.93	3.29	16.19
Stomatal conductance	44.43	5729.83 ***	231.72 ***	268.78 ***	75.42	51.87	31.86	38.58
Leaf relative water content	114.04	11,717.41 ***	208.98 ***	115.54 ^ns^	93.07	74.04	45.95	37.94
Chlorophyll a content	94.27	890.51 ***	3.93 ***	2.47 ***	1.72	11.93	9.42	21.02
Chlorophyll b content	175.53	1507.63 ***	8.01 ***	3.06 ^ns^	2.63	7.34	4.07	44.56
Total chlorophyll content	525.95	4715.53 ***	20.78 ***	7.53 ^ns^	6.98	19.27	13.49	29.98
Carotenoid content	2.41	3.29 ***	0.14 ^ns^	0.18 **	0.11	1.80	1.65	8.33
Shoot fresh biomass	0.94	28.18 ***	1.01 ***	0.28 ***	0.12	1.30	0.85	34.41
Shoot dry biomass	1.66	29.27 ***	0.05 ***	0.005 ^ns^	0.03	0.65	0.19	70.27

ns: non-significant; *, **, and *** significant at *p* < 0.05, *p* < 0.01, and *p* < 0.001, respectively. ^†^ *MC* mean values in the control condition, *MS* mean values in the water-deficit stress condition, *RC* Probability percentage change due to water-deficit stress compared with control.

**Table 2:** The estimated factor loading for the 95 investigated accessions was based on measured physiological and biochemical characters under control and water-deficit stress treatments.

Trait	Control Condition				Water-Deficit Stress Condition				
	PC1	PC2	PC3	PC4	PC1	PC2	PC3	PC4	PC5
SPAD	0.762	0.250	0.066	0.126	0.242	0.204	0.475	0.115	−0.488
Fo	0.494	−0.255	0.679	−0.243	0.432	0.562	−0.159	0.072	0.091
Fv/Fm	0.287	0.747	−0.106	0.044	−0.418	−0.380	0.710	0.171	−0.075
Fv/Fo	−0.529	0.328	−0.568	0.273	−0.553	−0.603	0.459	0.123	−0.026
SFW	0.229	0.720	−0.045	−0.206	0.002	0.635	0.539	0.112	0.062
SDW	−0.670	−0.385	−0.263	−0.060	−0.485	−0.562	−0.209	−0.080	0.025
RWC	0.052	0.403	0.267	−0.512	0.024	0.451	0.562	0.165	0.342
Chl a	0.786	−0.178	−0.081	0.450	0.815	−0.429	0.098	0.270	0.159
Chl b	0.757	−0.294	−0.498	−0.253	0.812	−0.412	0.239	−0.293	−0.002
Chl T	0.863	−0.276	−0.365	0.044	0.868	−0.448	0.186	−0.036	0.077
CAR	−0.057	0.109	0.499	0.797	0.043	−0.169	−0.344	0.886	0.168
GS	0.203	0.279	−0.252	0.169	−0.208	−0.057	0.152	−0.233	0.793
Eigenvalue	3.67	1.91	1.64	1.39	3.08	2.37	1.86	1.11	1.06
Variability (%)	30.61	15.89	13.69	11.60	25.68	19.78	15.49	9.23	8.86
Cumulative (%)	30.61	46.50	60.19	71.78	25.68	45.46	60.95	70.19	79.04

*SPAD* relative chlorophyll, *Gs* stomatal conductance (mmol m^−2^ s^−1^), *Fv/Fm* maximum quantum yield of PSII, *Fo* initial fluorescence, *Fv/Fo* maximum primary yield of PSII photochemistry, *Chl a* chlorophyll a content (μmol g^−1^ FW), *Chl b* chlorophyll b content (μmol g^−1^ FW), *Chl T* total chlorophyll content (μmol g^−1^ FW), *CAR* carotenoid content (μmol g^−1^ FW), *RWC* leaf relative water content, *SFB* shoot fresh biomass (g plant^−1^), and *SDB* shoot dry biomass (g plant^−1^).

**Table 3:** Detailed information about the used SSR primers along with the results of estimated informativeness parameters for each of them.

Primer	Chromosome Position	Sequence (5′–3′)		AT	N	H	PIC	Rp	MI
Xgwm-16	5D	F	GCTTGGACTAGCTAGAGTATCATAC	62.8	2	0.49	0.37	1.18	0.74
		R	CAATCTTCAATTCTGTCGCACGG						
Xgwm-44	7D	F	GTTGAGCTTTTCAGTTCGGC	59.9	2	0.35	0.29	1.57	0.58
		R	ACTGGCATCCACTGAGCTG						
Xgwm-111	7D	F	TCTGTAGGCTCTCTCCGACTG	59.5	2	0.18	0.16	1.86	0.32
		R	ACCTGATCAGATCCCACTCG						
Xgwm-121	5D & 7D	F	TCCTCTACAAACAAACACAC	54.3	2	0.49	0.37	1.11	0.98
		R	CTCGCAACTAGAGGTGTATG						
Xgwm-271	5D	F	CAAGATCGTGGAGCCAGC	58.5	2	0.43	0.34	1.43	0.74
		R	AGCTGCTAGCTTTTGGGACA						
Xgwm-272	5D	F	TGCTCTTTGGCGAATATATGG	55.9	2	0.25	0.21	1.75	0.68
		R	GTTCAAAACAAATTAAAAGGCCC						
Xgwm-292	5D	F	TCACCGTGGTCACCGAC	59.3	2	0.41	0.33	1.52	0.42
		R	CCACCGAGCCGATAATGTAC						
Xgwm-296	2D	F	AATTCAACCTACCAATCTCTG	55.6	2	0.48	0.36	1.26	0.66
		R	GCCTAATAAACTGAAAACGAG						
Xgwm-301	2D	F	GAGGAGTAAGACACATGCCC	59.5	2	0.49	0.37	1.25	0.72
		R	GTGGCTGGAGATTCAGGTTC						
Xgwm-325	6D	F	TTTCTTCTGTCGTTCTCTTCCC	69.3	2	0.49	0.37	1.03	0.74
		R	TTTTTACGCGTCAACGACG						
Xgwm-349	2D	F	GGCTTCCAGAAAACAACAGG	59.5	2	0.49	0.37	1.28	0.74
		R	ATCGGTGCGTACCATCCTAC						
Xgwm-382	2D	F	GTCAGATAACGCCGTCCAAT	59.2	2	0.48	0.36	1.20	0.74
		R	CTACGTGCACCACCATTTTG						
Xgwm-455	2D	F	ATTCGGTTCGCTAGCTACCA	56	2	0.49	0.37	1.22	0.72
		R	ACGGAGAGCAACCTGCC						
Xgwm-469	6D	F	CAACTCAGTGCTCACACAACG	63.5	2	0.50	0.37	1.04	0.74
		R	CGATAACCACTCATCCACACC						
Xgwm-515	2D	F	AACACAATGGCAAATGCAGA	60	2	0.46	0.35	1.34	0.70
		R	CCTTCCTAGTAAGTGTGCCTCA						
Xgwm-565	5D	F	GCGTCAGATATGCCTACCTAGG	62.1	2	0.30	0.26	1.69	0.52
		R	AGTGAGTTAGCCCTGAGCCA						
Xgwm-583	5D	F	TTCACACCCAACCAATAGCA	59.3	2	0.50	0.37	1.04	0.74
		R	TCTAGGCAGACACATGCCTG						
Xgwm-608	2D & 4D	F	ACATTGTGTGTGCGGCC	60.4	2	0.50	0.37	1.02	0.74
		R	GATCCCTCTCCGCTAGAAGC						
Xgwm-624	4D	F	TTGATATTAAATCTCTCTATGTG	51.3	2	0.49	0.38	1.14	0.76
		R	AATTTTATTTGAGCTATGCG						
Xgwm-639	5D	F	CTCTCTCCATTCGGTTTTCC	59.5	1	0	0	0	0
		R	CATGCCCCCCTTTTCTG						
Xgwm-157	2D	F	GTCGTCGCGGTAAGCTTG	60	2	0.50	0.37	1.05	0.74
		R	GAGTGAACACACGAGGCTTG						
Xgwm-212	5D	F	AAGCAACATTTGCTGCAATG	60	2	0.38	0.30	1.56	0.60
		R	TGCAGTTAACTTGTTGAAAGGA						
Xgwm-232	1D	F	ATCTCAACGGCAAGCCG	55	2	0.15	0.14	1.88	0.28
		R	CTGATGCAAGCAATCCACC						
Xgwm-311	2D	F	TCACGTGGAAGACGCTCC	60	2	0.46	0.35	1.31	0.70
		R	CTACGTGCACCACCATTTTG						
Xgwm-484	2D	F	ACATCGCTCTTCACAAACCC	55	2	0.49	0.37	1.15	0.74
		R	AGTTCCGGTCATGGCTAGG						
	Mean				1.96	0.41	0.32	1.27	0.80

AT, N, H, PIC, MI, and Rp indicated annealing temperature, the number of amplified alleles, gene diversity, polymorphism information content, marker index, and resolving power, respectively.

**Table 4:** Summary of estimated genetic variation indices in two *Ae. tauschii* and *T. aestivum* species.

Genetic Variation Parameter	*Ae. tauschii* (n = 48)	*T. aestivum* (n = 47)	Variation between Species	Variation within Species
Number of observed alleles (Na)	1.87 ± 0.05	1.65 ± 0.09	14%	86%
Number of effective alleles (Ne)	1.66 ± 0.04	1.47 ± 0.09		
Shannon’s information index (I)	0.53 ± 0.03	0.39 ± 0.05		
Nei’s genetic diversity (He)	0.37 ± 0.02	0.27 ± 0.04		
Percentage polymorphism loci (PPL)	89.8	73.47		

**Table 5:** Summary of marker–trait association analysis in *Ae. tauschii* and *T. aestivum* species under control and water-deficit stress conditions.

Trait	Control Condition			Trait	Water-Deficit Stress Condition		
	Marker	*p*-Value	*R* ^2^		Marker	*p*-Value	*R* ^2^
CAR	*Xgwm-121*	0.004	10.884	CAR	*Xgwm-121*	0.006	13.782
CAR	*Xgwm-271*	0.003	11.886	CAR	*Xgwm-271*	0.013	14.508
Chla	*Xgwm-111*	0.027	8.502	Chla	*Xgwm-111*	0.011	10.496
Chla	*Xgwm-44*	0.039	9.141	Chla	*Xgwm-44*	0.015	11.426
Chla	*Xgwm-455*	0.025	9.465	Chla	*Xgwm-455*	0.018	12.644
Chlb	*Xgwm-111*	0.975	8.053	Chlb	*Xgwm-111*	0.006	12.553
Chlb	*Xgwm-44*	0.876	9.281	Chlb	*Xgwm-44*	0.001	11.596
Chlb	*Xgwm-455*	0.014	10.334	Chlb	*Xgwm-455*	0.076	11.580
Chlb	*Xgwm-484*	0.045	6.789	Chlt	*Xgwm-111*	0.008	15.776
Chlt	*Xgwm-272*	0.042	6.052	Chlt	*Xgwm-44*	0.003	10.566
Chlt	*Xgwm-325*	0.039	6.180	Chlt	*Xgwm-455*	0.006	16.554
Chlt	*Xgwm-455*	0.037	8.785	Fo	*Xgwm-271*	0.035	8.418
Fo	*Xgwm-271*	0.006	15.214	Fo	*Xgwm-455*	0.148	11.292
Fv/Fm	*Xgwm-272*	0.005	10.554	Fv/Fm	*Xgwm-272*	0.012	14.748
Fv/Fm	*Xgwm-292*	0.008	9.742	Fv/Fm	*Xgwm-292*	0.047	16.037
Fv/Fo	*Xgwm-292*	0.020	8.676	Fv/Fo	*Xgwm-272*	0.046	5.242
Fv/Fo	*Xgwm-484*	0.009	16.919	Fv/Fo	*Xgwm-292*	0.047	5.924
Gs	*Xgwm-16*	0.020	8.599	Gs	*Xgwm-272*	0.008	14.198
Gs	*Xgwm-272*	0.020	12.427	RWC	*Xgwm-292*	0.040	8.877
RWC	*Xgwm-232*	0.049	6.490	RWC	*Xgwm-296*	0.037	9.216
RWC	*Xgwm-484*	0.025	5.271	RWC	*Xgwm-301*	0.022	10.017
SDW	*Xgwm-484*	0.045	5.614	SDW	*Xgwm-565*	0.040	7.125
SDW	*Xgwm-565*	0.039	6.325	SDW	*Xgwm-582*	0.047	6.775
SDW	*Xgwm-582*	0.030	6.887	SFW	*Xgwm-232*	0.016	8.761
SFW	*Xgwm-455*	0.015	9.750	SFW	*Xgwm-484*	0.019	9.397
SFW	*Xgwm-484*	0.012	8.587	SPAD	*Xgwm-111*	0.057	9.644
SPAD	*Xgwm-565*	0.011	9.117	SPAD	*Xgwm-44*	0.020	9.612
SPAD	*Xgwm-582*	0.014	8.613				

R^2^ indicates the coefficient of determination. *SPAD* relative chlorophyll, *Chl a* chlorophyll a content, *Chl b* chlorophyll b content, *Chl T* total chlorophyll content, *CAR* carotenoid content, *Fv/Fm* maximum quantum yield of PSII, *Fv/Fo* maximum primary yield of PSII photochemistry, *Fo* initial fluorescence, *Gs* stomatal conductance, *RWC* leaf relative water content, *SFB* shoot fresh biomass, and *SDB* shoot dry biomass.

## Data Availability

Not applicable.

## Supplementary Materials

The following supporting information can be downloaded at: https://www.mdpi.com/article/10.3390/biotech12010003/s1, Table S1: The passport of the 95 investigated *Ae. tauschii* and *T. aestivum* accessions.
