# Supplementary material for: Molecular and Physiological Variability in Bread Wheat and Its Wild Relative (Aegilops tauschii Coss.) Species under Water-Deficit Stress Conditions
Source: BioTech (Basel). 2022 Dec 28;12(1):3. doi: 10.3390/biotech12010003 (PMC9844422; doi:10.3390/biotech12010003)
Supplement: Supplementary file 1 [file biotech-12-00003-s001.zip › biotech-2040719-supplementary.pdf]

BioTech

Article

Molecular and Physiological Variability in Bread Wheat and Its Wild Relative (*Aegilops tauschii* Coss.) Species under Water-Deficit Stress Conditions

Zahra Khodadadi, Mansoor Omidi , Alireza Etminan, Asa Ebrahimi and Alireza Pour-Aboughadareh

Table S1. The passport of the 95 investigated *Ae. tauschii* and *T. aestivum* accessions.

No.	Gene bank codes	Species	No.	Gene bank codes	Species	No.	Gene bank codes	Species
1	IUGB-00133	*T. aestivum*	33	IUGB-00578	*T. aestivum*	65	IUGB-00366	*Ae. tauschii*
2	IUGB-00134	*T. aestivum*	34	IUGB-00602	*T. aestivum*	66	IUGB-00369	*Ae. tauschii*
3	IUGB-00264	*T. aestivum*	35	IUGB-00586	*T. aestivum*	67	IUGB-00402	*Ae. tauschii*
4	IUGB-00447	*T. aestivum*	36	IUGB-00598	*T. aestivum*	68	IUGB-00151	*Ae. tauschii*
5	IUGB-00453	*T. aestivum*	37	IUGB-00515	*T. aestivum*	69	IUGB-00291	*Ae. tauschii*
6	IUGB-00460	*T. aestivum*	38	IUGB-01847	*T. aestivum*	70	IUGB-00382	*Ae. tauschii*
7	IUGB-00480	*T. aestivum*	39	IUGB-00534	*T. aestivum*	71	IUGB-00238	*Ae. tauschii*
8	IUGB-00482	*T. aestivum*	40	IUGB-00613	*T. aestivum*	72	IUGB-00249	*Ae. tauschii*
9	IUGB-00485	*T. aestivum*	41	IUGB-00590	*T. aestivum*	73	IUGB-00367	*Ae. tauschii*
10	IUGB-00516	*T. aestivum*	42	IUGB-00606	*T. aestivum*	74	IUGB-00273	*Ae. tauschii*
11	IUGB-00911	*T. aestivum*	43	IUGB-00599	*T. aestivum*	75	IUGB-00274	*Ae. tauschii*
12	IUGB-01569	*T. aestivum*	44	IUGB-01840	*T. aestivum*	76	IUGB-00276	*Ae. tauschii*
13	IUGB-01635	*T. aestivum*	45	IUGB-00532	*T. aestivum*	77	IUGB-00279	*Ae. tauschii*
14	IUGB-01671	*T. aestivum*	46	IUGB-00580	*T. aestivum*	78	IUGB-00289	*Ae. tauschii*
15	IUGB-01695	*T. aestivum*	47	7499-2	*T. aestivum*	79	IUGB-00374	*Ae. tauschii*
16	IUGB-01696	*T. aestivum*	48	TN-01-0836	*Ae. tauschii*	80	IUGB-00383	*Ae. tauschii*
17	IUGB-00615	*T. aestivum*	49	IUGB-00020	*Ae. tauschii*	81	IUGB-00386	*Ae. tauschii*
18	IUGB-00597	*T. aestivum*	50	IUGB-00107	*Ae. tauschii*	82	IUGB-00396	*Ae. tauschii*
19	IUGB-00604	*T. aestivum*	51	IUGB-00164	*Ae. tauschii*	83	IUGB-00400	*Ae. tauschii*
20	IUGB-00603	*T. aestivum*	52	IUGB-00193	*Ae. tauschii*	84	IUGB-00401	*Ae. tauschii*
21	IUGB-00576	*T. aestivum*	53	IUGB-00196	*Ae. tauschii*	85	IUGB-00404	*Ae. tauschii*
22	IUGB-00618	*T. aestivum*	54	IUGB-00198	*Ae. tauschii*	86	IUGB-00405	*Ae. tauschii*
23	IUGB-01845	*T. aestivum*	55	IUGB-00039	*Ae. tauschii*	87	TN-01-1970	*Ae. tauschii*
24	IUGB-00518	*T. aestivum*	56	IUGB-00223	*Ae. tauschii*	88	TN-01-2120	*Ae. tauschii*
25	IUGB-00593	*T. aestivum*	57	IUGB-00224	*Ae. tauschii*	89	IUGB-00297	*Ae. tauschii*
26	IUGB-00570	*T. aestivum*	58	IUGB-00245	*Ae. tauschii*	90	IUGB-01746	*Ae. tauschii*
27	IUGB-00575	*T. aestivum*	59	IUGB-00247	*Ae. tauschii*	91	KC-50006	*Ae. tauschii*
28	IUGB-01846	*T. aestivum*	60	IUGB-00260	*Ae. tauschii*	92	KC-50084	*Ae. tauschii*
29	IUGBI-00577	*T. aestivum*	61	IUGB-00261	*Ae. tauschii*	93	TN-01-0312	*Ae. tauschii*
30	IUGBI-00589	*T. aestivum*	62	IUGB-00143	*Ae. tauschii*	94	TN-01-1559	*Ae. tauschii*
31	IUGB-00573	*T. aestivum*	63	IUGB-00325	*Ae. tauschii*	95	IUGB-00141	*Ae. tauschii*
32	IUGB-00600	*T. aestivum*	64	IUGB-00365	*Ae. tauschii*			
